# Determination of appropriate energy threshold range for accurate estimation of effective atomic number considering statistical uncertainty in photon‐counting techniques

**DOI:** 10.1002/acm2.70007

**Published:** 2025-02-20

**Authors:** Tomonobu Haba, Hiroaki Hayashi, Tsukasa Takahashi, Shota Naito, Yuichi Furukawa, Shuichiro Yamamoto, Natsumi Kimoto, Shigeki Kobayashi

**Affiliations:** ^1^ Faculty of Radiological Technology School of Medical Science Fujita Health University Aichi Japan; ^2^ College of Transdisciplinary Sciences for Innovation Kanazawa University Ishikawa Japan; ^3^ Department of Radiology Fujita Health University Hospital Aichi Japan; ^4^ Graduate School of Health Science Fujita Health University Aichi Japan; ^5^ JOB CORPORATION Kanagawa Japan; ^6^ Department of Radiological Science Faculty of Health Sciences Junshin Gakuen University Fukuoka Japan

**Keywords:** effective atomic number, energy threshold, material identification, Monte Carlo simulation, photon counting

## Abstract

**Purpose:**

The energy threshold is an important parameter for precise material identification employing photon‐counting techniques. However, in such applications, the appropriate energy threshold has not been clarified. Therefore, we aimed to determine the appropriate energy threshold range for precise material identification, focusing on effective atomic number (*Z*) values as an index.

**Methods:**

The atomic number was estimated using a previously proposed algorithm and Monte Carlo simulations. This algorithm included three steps: calculating the attenuation factor from the incident photon counts on a photon‐counting detector, correcting the beam‐hardening effects, and estimating the atomic number from the attenuation factor index using the calibration curve. Monte Carlo simulations were performed to add Poisson noise to an ideal x‐ray spectrum. The total number of incident x‐rays was set in the range of 10^3^–10^6^. The x‐ray spectra were generated at tube voltages of 50–120 kV. Polymethyl methacrylate (*Z* = 6.5) and aluminum (*Z* = 13) were used for the analysis. The energy threshold was varied at intervals of 1 keV to estimate the atomic number. We evaluated the appropriate energy threshold range for accurately estimating the atomic number using the obtained atomic number data and statistical uncertainty under various conditions.

**Results:**

The appropriate energy threshold range was found to be 31–38 keV for a tube voltage range of 50–120 kV. At this energy threshold, the atomic number can be estimated within an accuracy of ± 0.7 at 10^5^ counts for the atomic number range of 6.5 (PMMA) to 13 (Al).

**Conclusions:**

We found the appropriate energy threshold range. The findings of this study are expected to be useful for appropriately setting the energy threshold during precise material identification using photon‐counting detectors for clinical applications.

## 1 INTRODUCTION

Photon‐counting detectors have attracted considerable attention as novel imaging detectors for x‐ray imaging diagnostics.^1^, ^2^ These detectors can analyze x‐rays individually and discriminate the energy of each x‐ray using several energy bins.^3^ Conversely, energy‐integrating detectors, typically used in x‐ray imaging diagnostics, produce detector signals proportional to the total absorbed x‐ray energies.^3^ Several studies have reported that x‐ray images using photon‐counting detectors exhibit characteristics such as higher spatial resolution,^4^, ^5^ improved contrast‐to‐noise ratio,^6^, ^7^ low‐dose imaging,^8^, ^9^ and material identification,^10^, ^11^, ^12^ compared to energy‐integrating detectors.

Material identification using photon‐counting techniques has been reported in several modalities, including x‐ray computed tomography (CT),^10^ mammography,^11^ and general x‐ray examinations.^12^ Holly et al. used clinical photon‐counting CT to examine x‐ray CT images for assessing the performance of fat and iron quantification and compared it to dual‐energy CT, magnetic resonance imaging, and magnetic resonance spectroscopy.^10^ Sasaki et al. estimated the mammary gland composition for distinguishing between breast tumors and mammary gland tissues using a cadmium telluride (CdTe) series photon‐counting detector.^11^ Kimoto et al. proposed a high‐accuracy material identification method for correcting both the beam‐hardening effect and detector response using a CZT photon‐counting detector.^12^ These detectors enable material identification because they provide information on the multiple linear attenuation coefficients at the corresponding energy bins for any material.^1^ Various material identification methods have been reported, including effective atomic number (Z_eff_) imaging,^12^ soft‐tissue and bone imaging,^13^ and K‐edge imaging.^14^ Asahara et al. demonstrated the utility of Z_eff_ imaging in forensic dental identification using photon‐counting CT (PC‐CT).^15^ Their study showed that Z_eff_ values can be measured with a systematic error of 0.2–0.4 across multiple modalities, including PC‐CT, dual‐energy CT, and photon‐counting‐scanogram systems (general x‐ray examinations). They proposed that Z_eff_ values could serve as a quantitative diagnostic index independent of the imaging modality. In alignment with this approach, we focused on employing effective atomic number values as an index for material identification.

The ratio of two linear attenuation coefficients facilitates the identification of the atomic number value.^2^, ^15^ Thus, the accuracy of the linear attenuation coefficients for each energy bin is important for the precise estimation of the atomic number. The energy bins are determined using energy thresholds in the x‐ray spectrum. The performance of material identification is dependent on the position of the energy thresholds.^1^ Therefore, appropriate energy thresholds must be determined for precise material identification.

However, appropriate energy thresholds for precise material identification have not yet been clarified, despite several studies proposing methods to set energy thresholds.^16^, ^17^, ^18^ Schmidt, for instance, adopted five energy bins to improve contrast‐to‐noise ratio (CNR) for energy‐resolved CT but noted that no attempt was made to optimize the bin widths (i.e., energy thresholds) other than for the lowest energy bin.^16^, ^17^ Similarly, Nakajima et al. evaluated conditions for energy thresholds to accurately estimate mammary gland composition using a CdTe series photon‐counting detector.^18^ Using three energy bins at 50 kV, they concluded that increasing the width of the middle‐energy bin is beneficial. Nonetheless, their study evaluated only four combinations of energy thresholds, leaving the appropriate energy thresholds undefined.

Hence, in this study, we aimed to determine the appropriate energy threshold range for precise material identification (effective atomic number values) in general x‐ray examinations. Kimoto et al. demonstrated that the accuracy of effective atomic number estimation improves as the incident x‐ray count increases.^19^ The number of incident x‐ray counts contributes to statistical uncertainties in x‐ray imaging. Therefore, we focused on the possibility that statistical uncertainties were an important factor in determining the appropriate energy threshold. Specifically, the energy threshold changes the ratio of incident x‐ray counts in each energy bin, affecting the accuracy of effective atomic number estimation. We calculated the atomic number values and assessed statistical uncertainty using Monte Carlo simulations and Kimoto's algorithm.^12^ We used two energy bins to calculate atomic number values because this was sufficient for substances containing only light elements, such as human tissue, for non‐contrast enhanced images.^2^ This point is explained in detail as follows. In the diagnostic x‐ray energy range of 30–120 keV, the main interactions are the photoelectric effect and Compton scattering. Since light elements that compose the human body do not have a K‐edge in this energy range, two energy bins are sufficient for determining the effective atomic number. If the K‐edge is in this energy range, the mass attenuation coefficient μ/ρ becomes discontinuous to x‐ray energy, and more than three energy bins are needed for material identification. This applies, for example, to the use of iodine (*Z* = 53) or gadolinium (*Z* = 64).

The paper is organized as follows. We first describe Kimoto's algorithm for estimating the atomic number using photon‐counting techniques. Next, we report on the Monte Carlo simulations performed to calculate the atomic number and statistical uncertainty. Finally, we determine the appropriate energy threshold range. This study provides valuable knowledge on setting the appropriate energy threshold for precise material identification using photon‐counting techniques for a range of light elements (*Z* = 6.5–13).

## 2 MATERIALS AND METHODS

### 2.1 Algorithm for estimating atomic number *Z* using photon‐counting techniques

**FIGURE 1:**
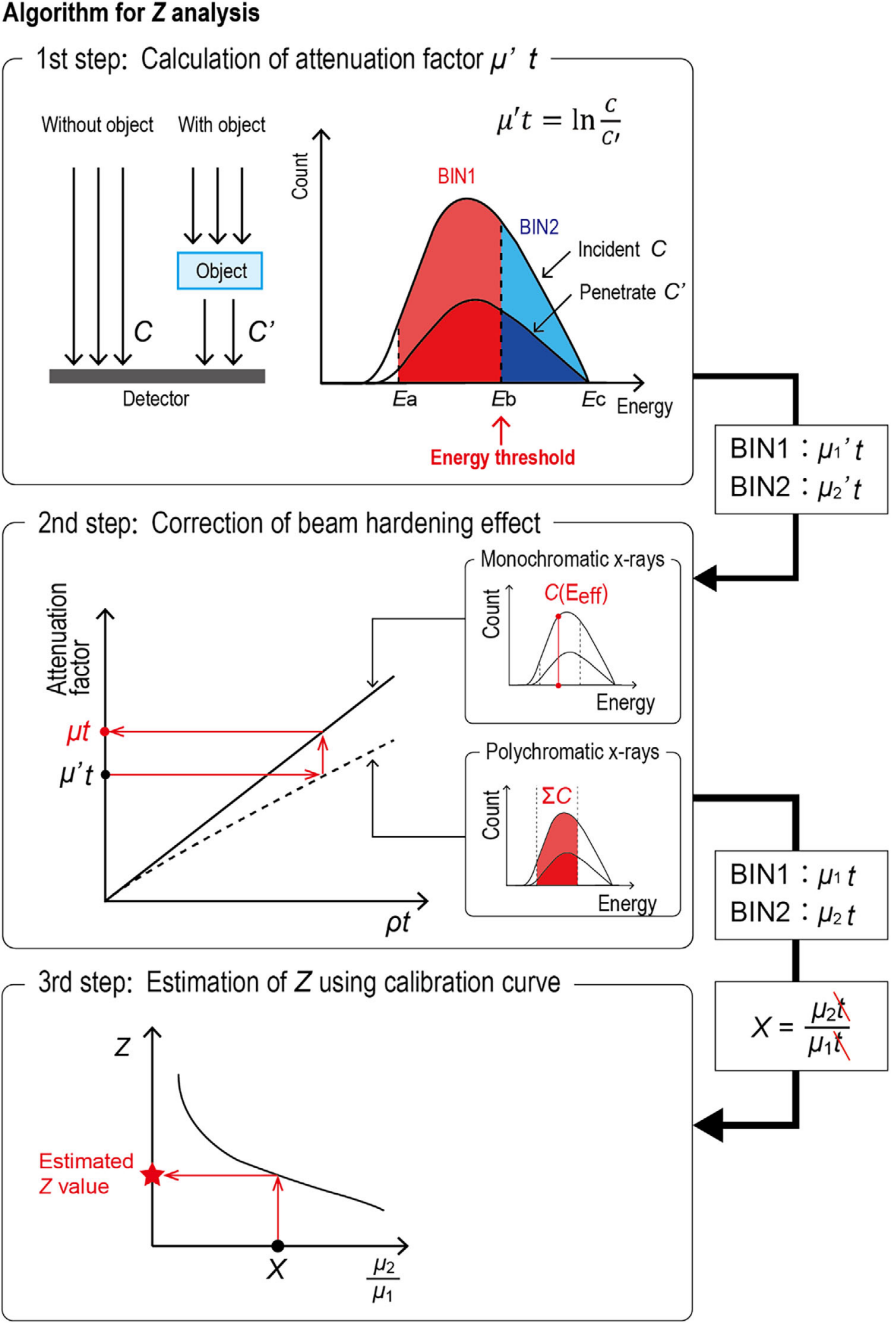
Schema of the algorithm for analyzing the atomic number *Z*. The algorithm is based on Kimoto's study^19^ and includes three steps: (1) calculation of the attenuation factor μ′*t*, (2) correction of the beam‐hardening effect, and (3) estimation of *Z* using the calibration curve. A detailed explanation of each step is described in the main text (Section 2.1).

The algorithm for estimating *Z* using photon‐counting techniques is shown in Figure 1. This algorithm is based on Kimoto's method,^19^, ^20^ which was previously reported, and includes three steps:

Step 1: Calculate the attenuation factor μ′*t* from the incident photon counts on the photon‐counting detector using

(1) $$\mu^{\prime }t=\ln\frac{C}{C^{\prime }},$$

where *C* and *C*′ represent the photon count passing through the air (without the object) and that after penetrating the object (with the object), respectively.

In this study, two energy bins were used (BIN1 and BIN2 for the low‐ and high‐energy bins, respectively). These energy bins were divided according to their energy thresholds *Eb*. The energy range of BIN1 was *Ea*–*Eb* keV, and that of BIN2 was *Eb*–*Ec* keV. The photon counts (*C* and *C*′) integrated in the energy range were obtained for each bin. Therefore, the μ′*t* was calculated for each bin (*μ_1_’t* for BIN1 and *μ_2_’t* for BIN2) based on Equation (1). These μ′*t* values were affected by the beam‐hardening effect,^21^ making beam‐hardening correction necessary.

Step 2: Perform beam‐hardening correction.^19^

The relationship between the attenuation factor (μ′*t* and *μt)* and mass thickness *ρt* (*ρ* and *t* represents the density and thickness of an object, respectively) was calculated in advance based on the database of x‐ray mass attenuation coefficients.^22^ In the center graph of Figure 1, the dotted curve line indicates the relationship between μ′*t* and *ρt*, whereas the solid linear line indicates the relationship between *μt* and *ρt*. μ′*t* was an attenuation factor affected by the beam‐hardening effect. Polychromatic x‐ray counts in the bin were used for calculating the μ′*t* curve line. Conversely, *μt* represents the attenuation factor unaffected by the beam‐hardening effect. Monochromatic x‐ray counts at the effective energy *E_eff_* in the bin were used for calculating the *μt* linear line. Using these two lines, the beam‐hardening correction was performed as follows. The *ρt* value corresponding to the μ′*t* value obtained in the first step was calculated using the polychromatic x‐ray curve (dotted curve line). Subsequently, the *μt* value corresponding to the *ρt* value was calculated using the monochromatic x‐ray curve (solid linear line). The flow of this procedure is indicated by the red arrows in Figure 1. This beam‐hardening correction process was performed for each bin. Thus, *μt* values were obtained for each bin (*μ_1_t* for BIN1 and *μ_2_t* for BIN2).

Step 3: Estimate the atomic numbers using a calibration curve.

The *μ_2_*/*μ_1_* value was adopted as the attenuation factor index. At the bottom graph of Figure 1, the vertical and horizontal axes of the calibration curve were the atomic number *Z* and attenuation factor index *μ_2_*/*μ_1_*, respectively. These relationships were calculated in advance based on the x‐ray mass attenuation coefficients.^22^ Using the calibration curve, the atomic number can be estimated as follows. The *μ_2_*/*μ_1_* value *X* of the object to be analyzed was calculated by dividing the *μ_2_t* value by the *μ_1_t* value obtained in the second step. The attenuation factor index facilitated the analysis of *Z* independent of the object thickness *t*. Consequently, the atomic number of the object was estimated using the calibration curve and *μ_2_*/*μ_1_* value *X*. The flow of this procedure is indicated by the red arrows in Figure 1.

### 2.2 Simulation method for estimating the atomic number *Z* and statistical uncertainty

#### 2.2.1 X‐ray spectra conditions

**FIGURE 2:**
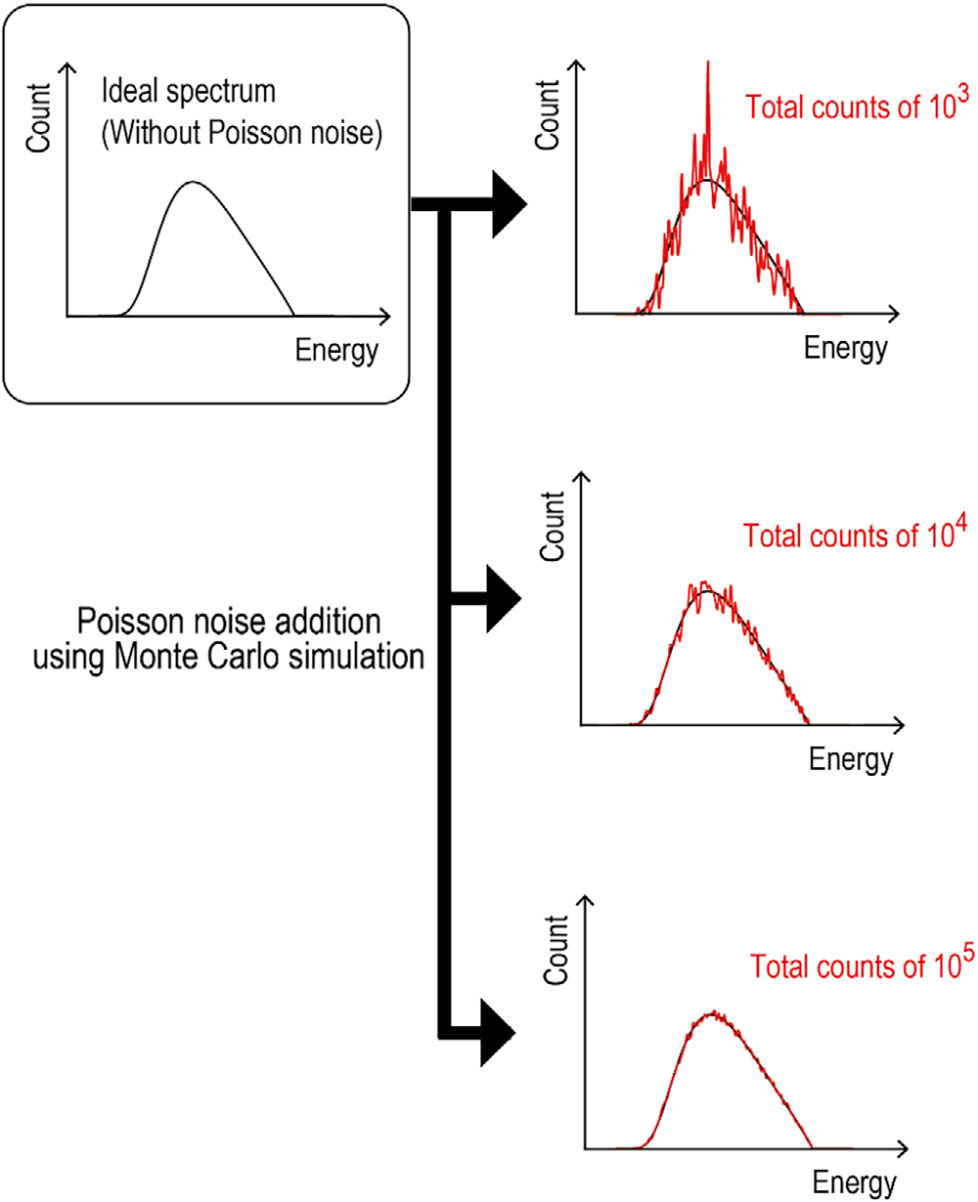
Schema of Poisson noise addition using Monte Carlo simulation. As examples, the x‐ray spectra with total counts of 10^3^, 10^4^, and 10^5^ are illustrated.

The x‐ray spectra were generated using Tucker's formula,^23^ which is a semi‐empirical model for generating the x‐ray spectra of the tungsten target. We set the x‐ray spectra at various tube voltages (50–120 kV) with a total filtration of 2.5 mm aluminum. The x‐ray spectrum has statistical fluctuations based on a Poisson distribution,^24^ and therefore, we added Poisson noise to the ideal x‐ray spectrum generated from Tucker's formula using a Monte Carlo simulation. Monte Carlo simulations were performed using Microsoft Excel and Visual Basic for Applications. In Excel, the RAND function was used to generate random numbers. The total number of incident x‐rays was set in the range of 10^3^–10^6^. A lower limit of 10^3^ counts was selected because statistical fluctuations become too significant for analysis below these counts. An upper limit of 10^6^ counts was chosen, similar to the count levels commonly used in current x‐ray diagnostics.^25^ Figure 2 shows the schema of Poisson noise addition using Monte Carlo simulation.

The method for dividing the two energy bins is as follows. In the first step shown in Figure 1, *Ea* is the lower limit value of BIN1. We set *Ea* to 20 keV because the typical lowest threshold for a photon‐counting detector is in the range of 20–25 keV,^1^, ^2^ where *Ec* represents the upper limit of BIN2. *Ec* was set to an energy equal to the x‐ray tube voltage. The two bins were divided by the energy threshold *Eb*, which was set to a value ranging from 21 to “*Ec −* 1” keV with an interval of 1 keV.

#### 2.2.2 Conditions of analysis materials

Polymethyl methacrylate (PMMA, *Z* = 6.5) and aluminum (Al, *Z *= 13) were used as materials in the analysis. PMMA and Al were selected because the atomic numbers of PMMA and Al are close to that of soft tissue (*Z* = 7) and bone (*Z* = 13), respectively, which comprise the human body.^22^, ^26^ An analysis of soft tissues and bones is a common practice in medical diagnosis.^3^ The thickness was set to 20, 40, 80, and 160 mm for PMMA and 2, 4, 8, and 16 mm for Al.

#### 2.2.3 Calculation of atomic number *Z* and statistical uncertainty

The atomic number value was calculated based on the aforementioned methodology using the algorithm described in Figure 1 under various conditions (different values of tube voltage, energy threshold, material type and thickness, and photon count). The simulation conditions are listed in Table 1. When Poisson noise was added, Monte Carlo simulations were performed five times to calculate the average value of the atomic number and statistical uncertainty (standard deviation [SD]). The simulations were performed for all combinations of these conditions. Further, the statistical uncertainty was calculated using the SD value in the Poisson distribution and error propagation formula^24^ for comparisons with the Monte Carlo simulation results. In the Poisson distribution, the SD for the counts can be determined using the square root of the counts (i.e., $\sigma \left(C\right)=\sqrt{C}$). Further, in Equation (1), the SD for an attenuation factor μ′*t* was calculated using the error propagation formula. Thereafter, the SD of the atomic number was calculated according to the algorithm shown in Figure 1.

**TABLE 1:** Simulation conditions for calculating the atomic number *Z* and statistical uncertainty. The simulations were performed under combinations of these conditions.

Parameters	Values
Tube voltage (kV)	50–120 at intervals of 10 kV
Energy threshold (keV)	21 to “*Ec* − 1” (*Ec*: A maximum energy of tube voltage)
Material type (thickness [mm])	PMMA (20, 40, 80, and 160)
	Al (2, 4, 8, and 16)
Photon count	Ideal (without Poisson noise)
	Poisson noise addition (total counts of 10^3^–10^6^)

### 2.3 Determination of appropriate energy threshold range for accurate estimation of the atomic number

To determine the appropriate energy threshold range, we first investigated the relationship between the estimation accuracy of atomic number and energy threshold with and without statistical uncertainty. Next, the energy threshold with the smallest statistical uncertainty in atomic number was determined as the appropriate energy threshold. Finally, the estimation accuracy of atomic number for appropriate or inappropriate energy threshold choices was evaluated over a range of photon counts of 10^3^–10^6^.

## 3 RESULTS

### 3.1 Relationship between the estimation accuracy of atomic number and energy threshold

**FIGURE 3:**
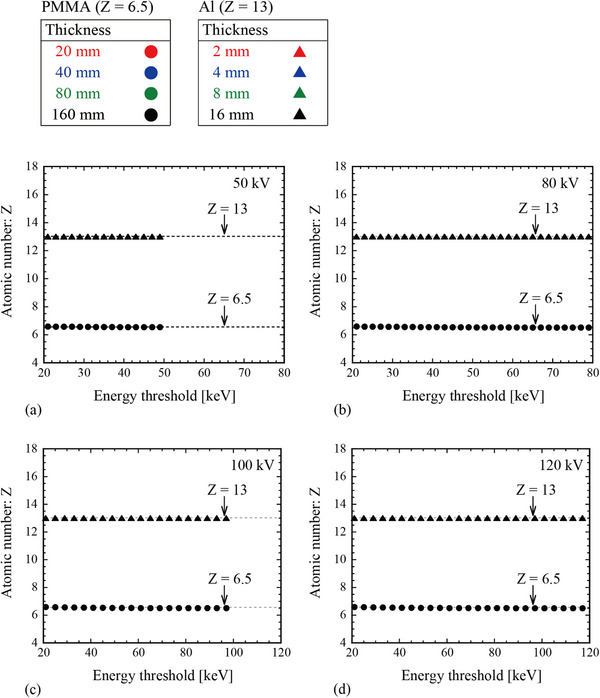
Relationship between the estimated atomic number value and energy threshold without statistical uncertainty (i.e., ideal x‐ray spectra). Various simulated conditions (tube voltage, material type, and material thickness) are considered. Symbol colors indicate the material thickness. The estimated atomic number values are consistent with the theoretical values (*Z* = 6.5 for PMMA and *Z* = 13 for Al), and therefore, the symbols overlapped. The results indicate that any energy threshold can be employed to accurately estimate the atomic number under ideal conditions with no statistical uncertainty.

Figure 3 shows the relationship between the estimated atomic number value and energy threshold without statistical uncertainty (i.e., assuming ideal x‐ray spectra). The data exhibited all combinations of the other parameters (tube voltage, and material type and thickness) listed in Table 1. The results indicate that the estimated atomic numbers were consistent with the theoretical values (*Z* = 6.5 for PMMA and *Z *= 13 for Al) at all energy thresholds under conditions with no statistical uncertainty.

**FIGURE 4:**
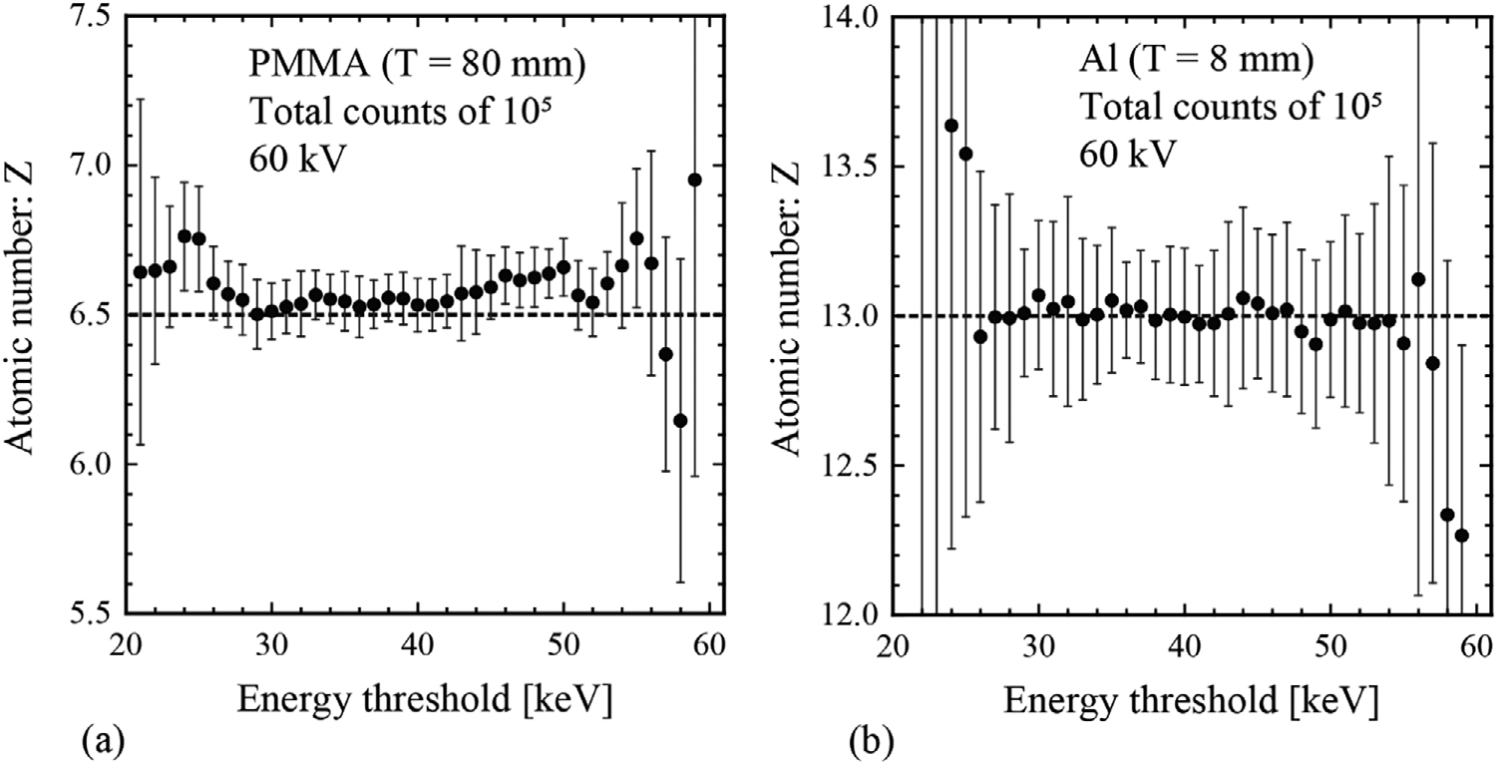
Relationship between the estimated atomic number value and energy threshold with statistical uncertainties (i.e., real x‐ray spectra). The following Monte Carlo simulation conditions are considered: photon counts of 10^5^, tube voltage of 60 kV, and material of PMMA (80 mm thickness) and Al (8 mm thickness). The closed circles and error bars show the averaged *Z* and SD values, respectively, obtained through five simulations. The dashed horizontal line indicates the theoretical values of PMMA and Al. Taking statistical uncertainties into account, the accuracy of atomic number estimation varies depending on the energy threshold.

Figure 4 shows the relationship between the estimated atomic number value and energy threshold with statistical uncertainty (i.e., for real x‐ray spectra) using the Monte Carlo simulations. In contrast to the results shown in Figure 3, the accuracy of atomic number estimation varied depending on the energy threshold in the presence of statistical uncertainty. This tendency was particularly pronounced when the energy threshold was set near the edge of the configurable range.

### 3.2 Determination of the appropriate energy threshold range for accurately estimating atomic numbers

**FIGURE 5:**
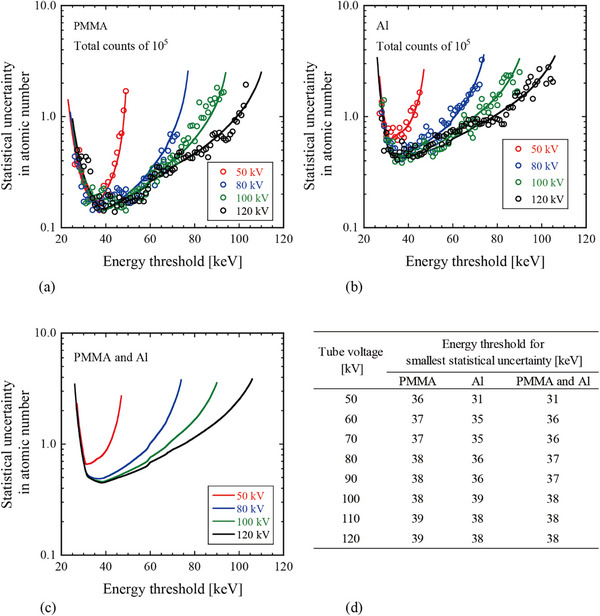
Relationship between the statistical uncertainty in the estimated atomic number and energy threshold. The data with the largest statistical uncertainty are plotted for all material thicknesses listed in Table 1 for (a) PMMA and (b) Al at each tube voltage. The total counts of incident x‐rays are set at 10^5^. The circle symbols indicate the Monte Carlo simulation results, and the solid lines indicate the results obtained by the error propagation formula with Poisson distribution. (c) Statistical uncertainty considering both PMMA and Al. (d) Energy threshold for smallest statistical uncertainty (i.e., appropriate energy threshold) at each tube voltage. The results indicate that the appropriate energy threshold range is 31–38 keV for tube voltages in the range of 50–120 kV.

Figure 5 shows the relationship between the statistical uncertainty in the estimated atomic number and energy threshold. Monte Carlo simulation and the error propagation formula with Poisson distribution were used for the analysis. The results of the two methods were consistent. When using the error propagation formula with the Poisson distribution, energy thresholds with the smallest statistical uncertainty in atomic number (i.e., appropriate energy threshold range) were determined in the range of 31–38 keV for tube voltages of 50–120 kV. At such energy thresholds, the atomic number can be estimated within an accuracy of ± 0.7 at 10^5^ counts for the atomic number range of 6.5 (PMMA) to 13 (Al).

### 3.3 Evaluation of the estimation accuracy of atomic number for appropriate or inappropriate energy threshold

**FIGURE 6:**
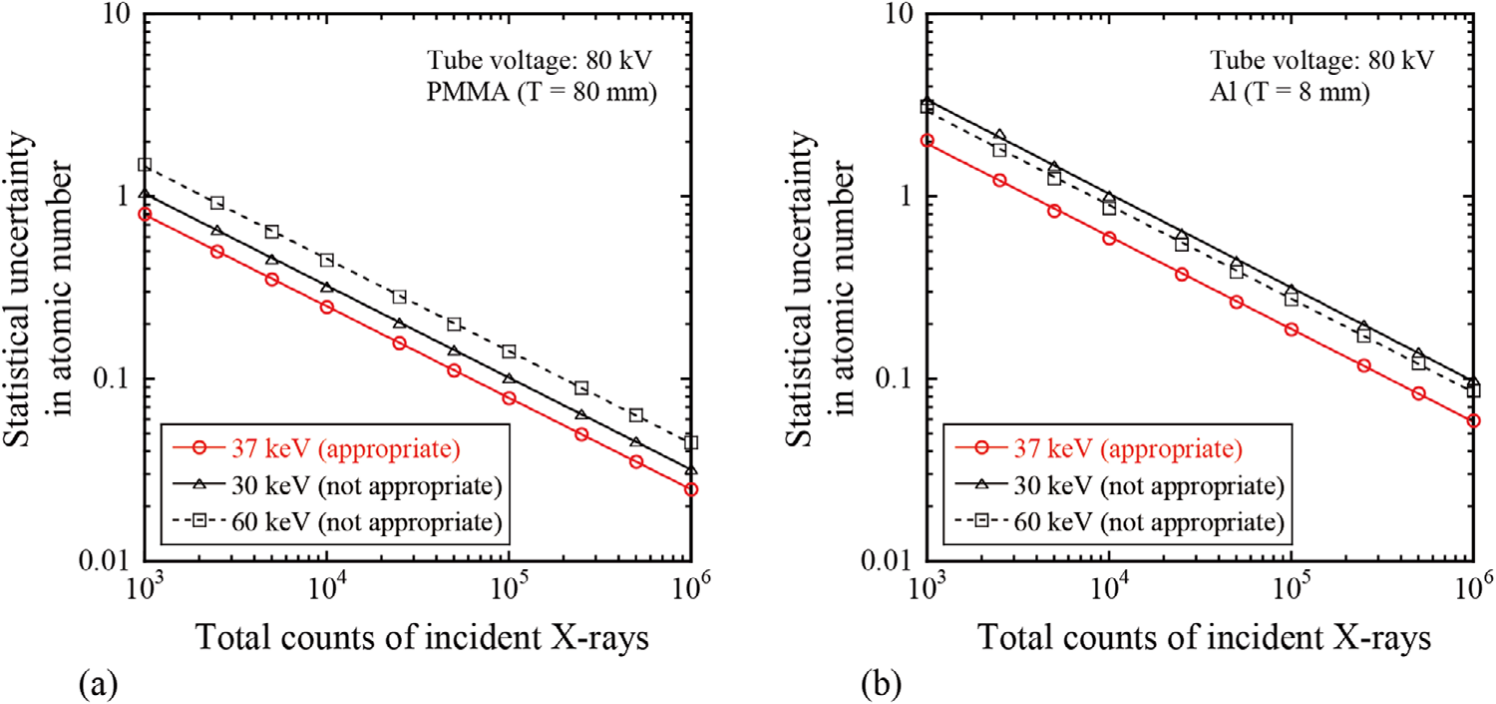
Relationship between statistical uncertainty in estimated atomic number and total counts of incident x‐rays under conditions with appropriate (e.g., 37 keV) and inappropriate (e.g., 30 or 60 keV) energy thresholds. The analysis conditions were tube voltage = 80 kV, object materials (a) PMMA (80 mm thickness) and (b) Al (8 mm thickness), and total incident x‐ray counts in the range of 10^3^–10^6^. The data are obtained by error propagation using a Poisson distribution. The results indicate that under the same photon count conditions, the atomic number can be estimated more accurately when the appropriate energy threshold is employed compared to the inappropriate energy threshold.

Figure 6 shows the relationship between the statistical uncertainty in the estimated atomic number and total counts of incident x‐rays under conditions of appropriate (e.g., 37 keV) or inappropriate (e.g., 30 or 60 keV) energy thresholds for the tube voltage of 80 kV. The statistical uncertainties in atomic number were lower within the appropriate energy threshold compared to the inappropriate energy threshold across all photon counts in the range of 10^3^–10^6^.

## 4 DISCUSSION

We proposed an appropriate energy threshold range for dividing the two energy bins to accurately estimate the atomic number using photon‐counting techniques.

Figure 3 shows that the estimated atomic numbers were consistent with the theoretical values at all energy thresholds for conditions with no statistical uncertainty (i.e., assuming ideal x‐ray spectra). This result indicated that all energy thresholds are appropriate for estimating atomic number if statistical uncertainties are not considered. However, in practical applications using x‐rays, statistical uncertainties must be considered because they are inherent in the measurement process and cannot be overlooked. Figure 4 shows that the estimated atomic numbers were not consistent with the theoretical values when statistical uncertainties attributed to photon counts were added. This tendency was strongly observed when the energy threshold was set near the edge of the configurable range. Thus, we found that the statistical uncertainty caused by the number of photons is an important factor in determining the appropriate energy thresholds for the accurate estimation of the atomic number.

We evaluated the appropriate energy threshold range for dividing the two bins to precisely calculate the atomic number, as shown in Figure 5. The appropriate energy threshold range was 31–38 keV for tube voltages in the range of 50–120 kV. At this energy threshold, the atomic number can be estimated within an accuracy of ±0.7 at 10^5^ counts for the atomic number range of 6.5 (PMMA) to 13 (Al). Kimoto et al. utilized a photon‐counting scanogram imaging system (JOB CORPORATION, Kanagawa, Japan) to calculate the atomic number.^12^ They achieved an estimation accuracy of ±0.5 for incident x‐ray counts of 1.6 × 10^5^. This result aligns well with our findings. The accuracy of the estimated atomic number can be improved by increasing the photon count (as shown in Figure 6); however, the appropriate energy threshold range does not change because the amount of statistical uncertainties increases or decreases with the photon count as well at all energy thresholds (both appropriate and inappropriate energy thresholds). Therefore, by setting an appropriate energy threshold (31–38 keV), users can efficiently collect the necessary number of photons to achieve the required atomic number estimation accuracy for each specific clinical application. This results in a lower patient dose, as images can be generated using fewer photons, that is, with reduced x‐ray intensity.

The limitations of this study include the following: (1) The effect of the detector response was not included in the analysis of the appropriate energy threshold range. The detector response included factors such as detector materials,^3^, ^27^, ^28^ charge sharing effect,^29^, ^30^, ^31^ and energy resolution.^32^ Three main types of semiconductor detector materials have been proposed^1^: CdTe,^33^ CZT,^34^ and silicon (Si).^28^ The detector response affects the number of photons counted in each energy bin for a given spectrum, and the appropriate energy threshold range may vary. However, as the detector response can be corrected using software^12^, ^31^ and hardware^35^ approaches, analysis using an x‐ray spectrum unaffected by the detector response is a meaningful initial step. (2) Scatter from the object was not included in the analysis. Scatter is another important phenomenon that affects the measured attenuation coefficients differently for each energy bin. However, the effect of scatter depends on the detector pixel size and irradiation field. Therefore, generalizing the effect and incorporating it when determining the appropriate energy threshold range is challenging. Currently, several studies have incorporated innovations to reduce scattered x‐rays; these involve employing scatter shields^36^ and optimizing the distance between the object and the detector.^21^ If the effects of detector response and scatter are taken into account in the simulation, the shape of the real x‐ray spectrum (including detector response and scatter) is expected to differ from that of the ideal x‐ray spectrum (not including detector response and scatter). This alters the number of photons (*C* and *C*′), which subsequently changes the attenuation factor μ′*t*, as demonstrated in Equation (1). In other words, the shape of the correction curve (dotted curve) would change in the center graph of Figure 1. We expect that a more realistic simulation model (including detector response and scatter) would influence the results for the appropriate energy threshold range. We plan to calculate the correction curve including the detector response and scatter using Monte Carlo simulation in future work. (3) The simulation results are not compared with those of experiments. The estimation accuracy of *Z* using the algorithm was already evaluated in the previous study.^12^ Therefore, in this study, experiments with various energy threshold changes, as shown in Figure 5, should be performed to validate our simulation results. However, our photon counting device enables only four conditions (a combination of tube voltage and energy threshold) to be set by the user. In addition, the simulation results cannot simply be compared to experimental results obtained from our photon counting device because the simulation results do not include the detector response and scatter. Therefore, comparing experimental results at this time with our limited resources is difficult. Based on the above circumstances, we performed this study using the simulation as a basic study for system development for the future. One advantage of simulation is that individual parameters can be verified independently. For example, it is difficult to verify the tube voltage characteristics alone because of the influence of pile‐up in experimental measurements. Although the results obtained in this study are limited to the ideal x‐ray spectrum (not including detector response and scatter), the method we have developed to determine the appropriate energy threshold range will be useful for studies in this field. A similar analysis could be used to determine the appropriate energy threshold range for individual applications. In the future, we would like to take the detector response and scatter into account in the simulation and verify the comparison with experimental results.

## 5 CONCLUSIONS

We determined the appropriate energy threshold range for photon‐counting techniques toward precise material identification in general x‐ray examinations. The results showed that the statistical uncertainty caused by the number of photons is an important factor in determining the appropriate energy thresholds. We found that the appropriate energy threshold range was 31–38 keV at tube voltages of 50–120 kV. The atomic number could be estimated within an accuracy of ±0.7 for incident x‐ray counts of 10^5^ upon setting the appropriate energy threshold range. The findings of this study can facilitate the development of material identification methods using photon‐counting techniques for clinical applications. In future work, we would like to simulate a more realistic simulation model (including detector response and scatter) and verify its comparison with experimental results for clinical applications.

## AUTHOR CONTRIBUTIONS

**Tomonobu Haba**: Data analysis; visualization; writing—original draft. **Hiroaki Hayashi**, **Shuichiro Yamamoto**, and **Natsumi Kimoto**: Methodology; visualization; writing—review & editing. **Tsukasa Takahashi**, **Shota Naito**, and **Yuichi Furukawa**: Writing—review & editing. **Shigeki Kobayashi**: Supervision; writing—review & editing.

## CONFLICT OF INTEREST STATEMENT

This was a collaborative study between the JOB CORPORATION and Fujita Health University. Shuichiro Yamamoto is an employee of the JOB CORPORATION.

## Data Availability

All data generated or analyzed during this study are included in this published article.
